# Recent Advances in the Application of Cucurbitacin B as an Anticancer Agent

**DOI:** 10.3390/ijms26168003

**Published:** 2025-08-19

**Authors:** Dongge Yin, Hongyue Chen, Shuting Lin, Yufei Sun, Xiaohong Jing, Rongrong Chang, Yang Feng, Xiaoxv Dong, Changhai Qu, Jian Ni, Xingbin Yin

**Affiliations:** School of Chinese Materia Medica, Beijing University of Chinese Medicine, Beijing 102488, China; 20230941515@bucm.edu.cn (D.Y.); 20240935229@bucm.edu.cn (H.C.); 20240935230@bucm.edu.cn (S.L.); 20230935210@bucm.edu.cn (Y.S.); 20230935212@bucm.edu.cn (X.J.); 20230935211@bucm.edu.cn (R.C.); 20240935283@bucm.edu.cn (Y.F.); dxiaoxv@163.com (X.D.); quchanghai@bucm.edu.cn (C.Q.)

**Keywords:** cucurbitacin B, cancer, toxicity, mechanism of action

## Abstract

Cucurbitacin B (CuB), a tetracyclic triterpenoid compound isolated from Cucurbitaceae plants, exhibits inhibitory effects on various tumor cells (e.g., liver, gastric, and colorectal cancer cells). Since the 1970s–1980s, cucurbitacin tablets containing CuB have been used as an adjuvant therapy for chronic hepatitis and primary liver cancer. CuB exerts anticancer effects through multiple mechanisms: inducing apoptosis, cell cycle arrest (G2/M or S phase), autophagy, and cytoskeleton disruption; inhibiting migration, invasion, and angiogenesis (via VEGF/FAK/MMP-9 and Wnt/β-catenin pathways); regulating metabolic reprogramming and immune responses; inducing pyroptosis, ferroptosis, and epigenetic changes; and reversing tumor drug resistance. These effects are associated with signaling pathways like JAK/STAT, PI3K/Akt/mTOR, and FOXM1-KIF20A. To improve its application potential, strategies such as structural modification (e.g., NO donor conjugation), combination therapy (with gemcitabine or cisplatin), and nanomaterial-based delivery (e.g., liposomes and exosome-mimicking nanoparticles) have been developed to enhance efficacy, reduce toxicity, and improve bioavailability. CuB shows broad-spectrum anticancer activity, but further research is needed to clarify the mechanisms underlying its cell-specific sensitivity and interactions with the immune system. This review systematically summarizes the physicochemical properties, anticancer mechanisms, and strategies for applying CuB and suggests future research directions, providing references for scientific research and clinical translation.

## 1. Introduction

Cucurbitacin B (CuB), a tetracyclic triterpenoid compound isolated from plants of the Cucurbitaceae family, is the most abundant of the cucurbitacins. CuB has shown promising efficacy against various cancers, including pancreatic cancer [1,2]. It exerts antitumor effects by suppressing proliferation, inhibiting migration and invasion, preventing angiogenesis, reprogramming metabolism, and enhancing immunity. However, due to its non-selective toxicity, excessive CuB is also toxic to normal cells. Therefore, strategies such as modifying the structure of CuB, combining it with other drugs, and utilizing nanomaterial-based delivery systems are often employed to reduce its toxicity. In this review, we systematically summarize the chemical structure, physicochemical properties, anticancer activities, and relevant applications of CuB, which may serve as a reference for its further development and clinical application.

## 2. Structure and Physicochemical Properties of CuB

**Figure 1:**
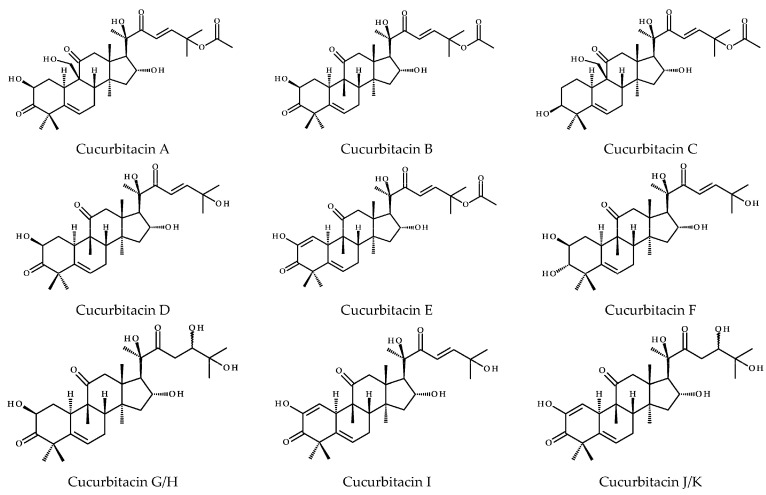

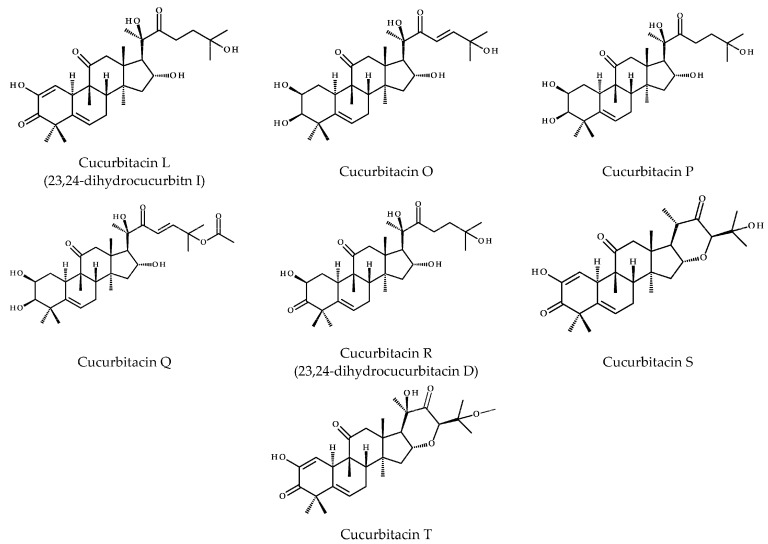
The structures of cucurbitacins A-T [6].

At least 100 species from 30 genera of the Cucurbitaceae family have been proven to contain cucurbitacins. The known cucurbitacins include cucurbitacins A, B, C, D, E, F, G, H, I, J, K, L, O, P, Q, R, S and T, the structures of which are shown in Figure 1. Among them, cucurbitacins B and D occur most frequently [3]. CuB (C_32_H_46_O_8_), present in 91% of the species in which cucurbitacins are found, is used as a natural tetracyclic triterpenoid medicine. Cucurbitacin is derived from the basic cucurbitacin ring skeleton, which is a type of triterpenoid hydrocarbon (IUPAC name: 19 (10→9β)-abeo-5α-lanostane). Modification with oxygen-containing groups and double bonds leads to the formation of many cucurbitacins with unique properties [4]. The skeletal structure of CuB (Figure 1) is 19 (10→9β)-abeo-10α-lanost-5-ene. In this structure, the methyl group at C-9 is in the β configuration, and the α-positioned hydrogen atom endows the structure with abnormal bioenergetic properties [5].

CuB is a white powder with a molecular weight of 558.7029 and a density of 1.0953 g/cm^3^. Some physicochemical properties of CuB are listed in Table 1. The data in the table are available at https://baike.baidu.com.

## 3. Pharmacological Properties of CuB as an Antitumor Agent

**Figure 2:**
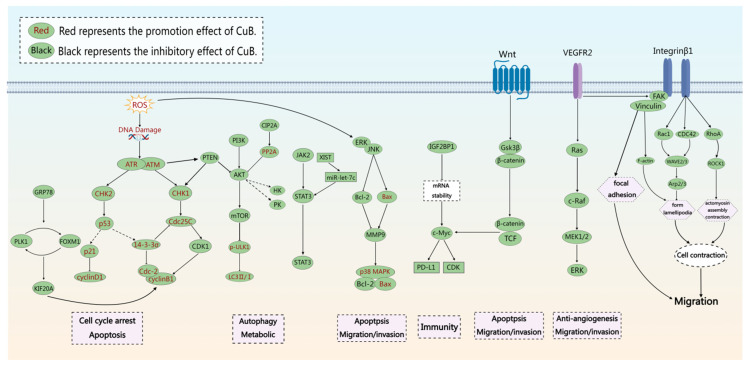
Molecular pathways involved in the anticancer pharmacological effects of CuB (both in vivo and in vitro). CuB exerts potent anticancer effects through regulation of FOXM1/KIF20A, PI3K/AKT, JAK/STAT3, CIP2A/PP2A, MAPK, Wnt/β-catenin, and VEGF/FAK signaling pathways (Created with MedPeer (medpeer.cn)).

Cucurbitacin F (CuF) has low-toxicity antibacterial and anti-inflammatory activities, and CuB is a derivative of CuF. CuB is found in plants of the Cucurbitaceae and Brassicaceae families. It is one of the most intensively studied compounds in its biological system and is highly toxic to various human cancer cells. We have summarized the literature on CuB from 2014 to 2024, and Table 2 shows its main anticancer mechanisms. The main antitumor pathways are illustrated in Figure 2.

## 4. Inhibit Cell Growth and Proliferation

Malignant tumors proliferate rapidly, facilitating their local infiltration or distant metastasis [44]. Previous studies indicate that promoting cell apoptosis and autophagy, inducing cell cycle arrest, and disrupting the cytoskeleton can effectively inhibit the proliferation and growth of cancer cells [45,46].

### 4.1. Promotion of Cell Apoptosis

Apoptosis is the main form of programmed cell death, playing a significant role in maintaining the homeostasis of normal tissues, cell differentiation, and development, especially in eliminating self-reactive or failed cells and controlling proliferative responses [47]. However, this effect is often inhibited in malignant tumors [48]. Therefore, promoting apoptosis is one of the main approaches to inhibiting tumor progression [49].

Reportedly, CuB induces apoptosis and cell cycle arrest in human prostate cancer cells by downregulating the Janus kinase (JAK)/Signal Transducers and Activators of Transcription (STAT) signaling cascade [23]. This signaling pathway plays an important role in regulating cell growth, differentiation, proliferation, and immune response [50]. Among the molecules in this pathway, STAT3 is a carcinogenic transcription factor closely related to tumorigenesis [51]. A previous study shows that CuB has no significant effect on the transcriptional level of STAT3 [22], but it can inhibit the expression of XIST and IL-6, enhance the expression of miR-let-7c, and then inhibit the phosphorylation of STAT3 and the IL-6/STAT3 pathway [25]. The integrin family is involved in the proliferation and metastasis of cancer cells; in particular, two major integrin heterodimers, ITGAVB3(αVβ3) and ITGA6B4(α6β4), have been found to play a specific role in breast cancer progression [8,52]. ITGB4 is overexpressed in breast cancer and interacts with HER2. CuB has also been proven to inhibit the expression of ITGA6B4, induce the expression of ITGB1 and ITGB3, and promote the apoptosis of breast cancer cells by inhibiting the HER2–integrin signaling pathway [8]. In addition, it was found that the promotion of apoptosis of human prostate cancer cells by CuB is related to ATP citrate lyase (ACLY) and can be offset by overexpression of ACLY [17]. Numerous studies have shown that CuB can also inhibit the PI3K/Akt pathway and further regulate the downstream apoptotic factors and proteins of the PI3K/Akt pathway, such as cytochrome c and apoptosis-inducing factor (AIF), the pro-apoptosis protein Bax, and the anti-apoptosis proteins Bcl-2 and Bcl-XL, leading to cancer cell apoptosis [36,38].

### 4.2. Induction of Cycle Arrest

The cell cycle is the basic process of a cell’s life, including proliferation and differentiation [53]. All somatic cells proliferate via the mitotic process, driven by progression through the cell cycle. Infinite cell proliferation contributes to tumor generation; therefore, inducing cell cycle arrest can be used as a target for cancer treatment [54,55].

STAT3 is a key signaling protein involved in cellular processes, such as cell cycle progression, migration, apoptosis, and angiogenesis [56,57]. CuB has been proven to exert antitumor effects by inhibiting STAT3. It can significantly inhibit the expression levels of Cyclin B1, CDK1, and CD133 and then induce cell cycle arrest at the G2/M phase [30], which is related to the downregulation of p-JAK2 and p-STAT3 [16,30]. However, CuB can also induce G2/M arrest through STAT3-independent mechanisms, which may be influenced by the dosage and treatment duration. For example, in BCBL-1 cells, p-STAT3 and STAT3 protein expression is slightly reduced by CuB (30 nM, 12 h) but suppressed by CuB (40 nM, 6 h). Nevertheless, CuB (30 nM, 6 h) does not significantly downregulate p-JAK2 or p-STAT3 but instead triggers G2/M arrest and apoptosis by inducing actin aggregation [39]. Furthermore, the signaling pathways involved in cell cycle arrest vary across different cell types. Guo et al. demonstrated that CuB (200 nM) induced DNA damage by increasing ROS levels [19]. In A549 cells, this ROS-dependent DNA damage leads to G2/M arrest in over half of the cell population through activation of the ATM-Chk1-Cdc25C-Cdk1 and p53-14-3-3-sigma cascades, independent of p-JAK2/p-STAT3 inhibition. Chan et al. showed that, beyond G2/M arrest, CuB (30 μM) induces S-phase arrest in BEL-7402 human hepatocellular carcinoma cells by downregulating cyclin D1 and Cdc2 [1]. In addition, KIF20A (also known as mitotic kinesin-like protein 2, MKlp2) transports chromosomes during mitosis and plays a key role in cell division [7]. PLK1 and FOXM1 are essential effectors required for KIF20A to exert its biological functions [15]. Studies have shown that CuB can target GRP78 (an upstream regulator of PLK) and then induce cell cycle arrest through the FOXM1-KIF20A pathway [15]. Interestingly, Li et al. proved that CuB significantly induced cell cycle arrest in the G2/M phase in Huh7, Hep3B, and Hepa1/6 cells without leading to apoptosis [34]. This phenomenon can be tuned by adding methyl propylamine (MPA), a DNA-protective agent.

It can be seen that the cell cycle arrest triggered by CuB predominantly manifests in the G2/M or S phase.

### 4.3. Induction of Cellular Autophagy

Autophagy, a lysosomal-dependent pathway of self-degradation, shows both tumor inhibition and carcinogenic activity in cancer, depending on the cell microenvironment and stages of tumor progression [58,59]. Therefore, autophagy, which has the dual effects of promoting either cell death or cell survival, appears to be a promising target for cancer treatment [60].

Autophagy dysfunction is associated with an increase in cancer progression due to DNA damage, chromosome instability, and oxidative stress [61]. A substantial amount of research has shown that CuB can induce autophagy in cancer cells, which is likely associated with ROS generation and the aggravation of DNA damage [9,29]. This autophagy induction manifests as the inhibition of the Akt/mTOR signaling pathway, accompanied by upregulation of the expression levels of autophagy-related proteins such as p-ULK1 and LC3II. In addition, Zhang et al. found that upon treatment with CuB, a significant number of layered structures and autophagic vacuoles were generated within HeLa cervical cancer cells [62]. In addition, the expression of microtubule-associated LC3 was significantly increased, indicating the activation of autophagy. This effect is achieved by enhancing the production of mitochondrial ROS and activating ERK and JNK.

In fact, apoptosis and autophagy are often intricately linked, with autophagy-related genes participating in the regulation of apoptosis [63]. The regulatory effect of CuB on apoptosis and autophagy needs further study.

### 4.4. Cytoskeleton Alterations

The cytoskeleton is a complex, dynamic biopolymer network composed of actin filaments, microtubules, and intermediate filaments. It is involved in maintaining cell morphology and mechanical support, migration, signal transduction, nutrient uptake, membrane and organelle trafficking, and cell division [64]. Numerous studies have demonstrated that inhibiting the migration and invasion of cancer cells and promoting cancer cell cycle arrest can be achieved by targeting the cytoskeleton [65,66,67].

Wang et al. studied the effects of CuB on microtubules and actin filaments in HeLa, MCF7, and U2OS cells using GFP markers and immunofluorescence staining [12]. The results showed that CuB affected the cytoskeleton by depolymerizing or aggregating actin filaments and inhibited the cell cycle in G2/M phase. However, some studies have shown that CuB indirectly increases actin aggregation and cofilin–actin rod formation by activating vasodilator-stimulated phosphoprotein (VASP), an effect mediated by the Ga13/RhoA/PKA pathway [14]. Liang et al. showed that CuB inhibits the adhesion and viscoelasticity of various cell lines, reduces cell abnormalities, and prevents the invasion and migration of malignant cells associated with the expression of F-actin/vimentin/FAK/vinculin [11]. These effects were also mediated by inhibiting the signaling pathway downstream of integrin, including the Rho GTPase family members RhoA, Rac1, and Cdc42.

In addition, studies have shown that the effect of CuB on the cytoskeleton is related to the JNK/c-Jun signaling pathway [68].

In summary, regardless of the mechanism, cytoskeleton alterations are typically accompanied by cell cycle arrest and reduced cell migration and invasion, which in turn inhibit tumor growth.

## 5. Inhibit Cancer Cell Migration and Invasion

Migration and invasion abilities are crucial characteristics and biomarkers of malignant tumors associated with a poor prognosis [69]. In addition to alterations in the cytoskeleton, the EMT phenomenon is also associated with a reduction in the migratory and invasive capabilities of cells [70].

CuB can inhibit the changes in cell morphology that occur in EMT, increase the protein expression of E-cadherin, reduce the protein expression of N-cadherin and vimentin, and thus inhibit cell migration and invasion. Further experiments have demonstrated that CuB inhibits the EMT of A549-GR cells through ROS and the PI3K/Akt/mTOR signaling pathway [24]. The PI3K/Akt signaling pathway has been proven to be involved in the regulation of TGF-β-induced EMT [71,72]. In addition, epidermal growth factor (EGF), Wnt, and integrin signaling pathways can all regulate EMT [73,74]. CuB exerts a discernible impact on all the aforementioned signaling pathways. For example, it inhibits the growth and invasion of gemcitabine-resistant non-small-cell lung cancer (GR-NSCLC) cells by inducing the lysosomal degradation of the EGF receptor (EGFR) and downregulating the CIP2A/PP2A/Akt signaling axis [26]. Matrix metalloproteinases (MMPs) can degrade various extracellular matrix proteins that may play important roles in cell proliferation, migration, angiogenesis, and apoptosis [75]. CuB has been shown to negatively modulate the VEGF/FAK/MMP-9 signaling axis, which consequently confers anti-metastatic and anti-angiogenic properties to cells [10]. Another study has shown that CuB can suppress the metastatic ability of NSCLC by inhibiting the Wnt/β-catenin signaling axis [21]. Moreover, Zhang et al. demonstrated that CuB inhibited pulmonary metastasis of colon cancer by suppressing M2 macrophage polarization via the JAK2/STAT3 signaling pathway [37].

Collectively, these findings suggest that CuB suppresses cell migration and invasion, which are associated with the cytoskeleton and EMT-related pathways.

### 5.1. Anti-Angiogenesis

As a vital factor for tumor growth and metastasis, angiogenesis is a hallmark of tumor malignancy, thus serving as a promising therapeutic target [76,77]. Angiogenesis is a complex process influenced by numerous receptors, growth factors, and cell interactions [78,79]. VEGF plays a crucial role in regulating angiogenesis and vascular permeability [80].

Sinha et al. showed that CuB significantly inhibited angiogenesis using semi-in vivo models such as the chorioallantoic membrane (CAM) and Matrigel plug assays [10]. Further experiments revealed that this effect was achieved by blocking VEGF-mediated tyrosine phosphorylation of FAK and inhibiting MMP activation. There are three types of VEGFRs: VEGFR1, VEGFR2, and VEGFR3. Among them, VEGFR2 is thought to be the primary mediator of angiogenesis [81]. Piao et al. showed that CuB dose-dependently inhibited VEGFR2 expression, reducing angiogenesis in vitro and in vivo [32]. Moreover, CuB can directly induce apoptosis of HUVECs via the mitochondrial apoptotic pathway during angiogenesis.

The formation of new blood vessels, a hallmark of tumor progression, provides cancer cells with oxygen and other nutrients to support their growth and metastasis [82]. Thus, angiogenesis inhibition is crucial for blocking cancer cell proliferation and metastasis, offering a novel approach to tumor progression prevention.

### 5.2. Improve Metabolic Reprogramming

Metabolic reprogramming can cause changes in metabolic phenotypes, alter energy metabolism in the tumor microenvironment, and subsequently promote tumor growth and proliferation [83,84]. Metabolic reprogramming mainly leads to abnormalities in lipid, glucose, and amino acid metabolism [85]. Glucose metabolism includes glycolysis, the tricarboxylic acid cycle (TCA), and the pentose phosphate pathway (PPP). Some malignancies, such as pancreatic ductal carcinoma, shift from oxidative phosphorylation to glycolysis for energy in the hypoxic tumor microenvironment. Pyruvate is oxidized to lactate rather than entering the TCA cycle [86]. Despite oxygen availability, excess pyruvate in the cytoplasm is primarily metabolized to lactate. This shift to aerobic glycolysis, known as the Warburg effect, is a hallmark of cancer cells [87].

CuB can induce DNA damage by increasing ROS levels and activate the PI3K/Akt/mTOR autophagy-related pathway. The PI3K/Akt/mTOR signaling pathway triggers the switch to aerobic glycolysis and orchestrates the reprogramming of glucose metabolism [88]. mTORC1 promotes glycolytic gene expression via the transcription factor Myc to regulate glucose transport and metabolism [89]. Previous studies indicate that LDHA and other metabolic effectors are downregulated by mTORC1 inhibition, and maximal cancer cell suppression occurs upon combination with a PI3K inhibitor [90]. Therefore, the inhibitory effect of CuB on cancer cells may be associated with the regulation of glucose metabolism reprogramming. Through UPLC-Q-TOF-MS/MS metabolomics, Ji et al. analyzed metabolite changes in mice treated with CuB [33]. Western blotting confirmed that CuB impacts lipid, amino acid, and glucose metabolism by altering the Akt/mTORC1 signaling pathway, hence decreasing tumor progression. Li et al. demonstrated that CuB can regulate the Akt/mTOR pathway to inhibit the activities of the key glycolytic enzymes HK and PK, reduce cellular glucose consumption as well as lactate and ATP production, and consequently suppress glycolysis and proliferation in HuCCT1 cells [91].

In conclusion, the inhibition of tumor progression by CuB through regulation of glucose metabolism reprogramming is also an effective therapeutic strategy, but further research is required to understand the specific mechanism.

### 5.3. The Impact on Immunity

Immunotherapy has emerged as a crucial approach to treating cancer and improving post-treatment prognosis [92,93]. Immunotherapy for cancer is typically based on the theory of the cancer immune cycle, which involves the enhancement of stimulatory immune factors and immune checkpoint inhibitors (ICIs) [94,95,96,97]. However, tumor cells evade immune surveillance through several immune escape mechanisms, including immunosuppression. Therefore, further development of immune antitumor drugs is an extremely urgent matter.

CuB and CuE appear to be the most intensively investigated agents in terms of the immune response. They exert significant effects on both innate and adaptive immune responses [98]. CuB has been proven to attenuate the expression of iNOS, COX-2, and MHC-II in macrophages [99]. It can suppress the polarization of M2 macrophages and induce the polarization of M1 macrophages in C57BL/6 mice [13]. Moreover, it promotes the differentiation of dendritic cells in patients with advanced lung cancer by suppressing the JAK2/STAT3 pathway, thereby enhancing clinical antitumor immunity [100]. In addition, numerous studies have shown that DNA damage caused by chemotherapeutic drugs can activate the innate immune response via the cGAS-STING pathway [101,102]. DNA damage induced by CuB can trigger cell autophagy and cell cycle arrest [29,34]. Yin et al. found that DNA damage induced by CuB can also promote the recruitment of γH2AX, inhibit the expression of ATR and the repair of broken DNA, and subsequently activate the cGAS pathway to trigger the innate immune response [41]. In addition, Liu et al. demonstrated that CuB can target IGF2BP1 and alter its structure [25]. Subsequently, it disrupts IGF2BP1 mRNA stability, resulting in the downregulation of immune-related genes such as Myc and KRAS, and recruits various immune-activating cells to the tumor site. Meanwhile, the expression of PD-L1 on the cell surface also decreases, ultimately improving the immunosuppressive tumor microenvironment.

In conclusion, CuB shows considerable potential in immunotherapy and plays a vital role in both innate and adaptive immunity. Nevertheless, the exploration of CuB’s role in regulating tumor immune responses remains limited, and it is worthy of further investigation.

### 5.4. Other Mechanisms

In addition to the aforementioned mechanisms, CuB can also inhibit tumor progression by inducing pyroptosis and ferroptosis, causing epigenetic changes, and improving drug resistance.

Pyroptosis is a form of programmed cell death (PCD) accompanied by an inflammatory response [103]. It is characterized by cell swelling and the release of cytokines such as IL-1β, IL-18, and HMGB1. Yuan et al. found that CuB induces pyroptosis in NSCLC by activating the NLRP3 inflammasome, promoting ROS production, Tom20 accumulation, and Ca^2+^ aggregation [27].

In addition, ferroptosis is a newly discovered form of PCD, which is mainly characterized by intracellular iron overload and accumulation of iron-dependent lipid peroxides [104]. Research has shown that CuB can induce ferroptosis by increasing lipid peroxidation and reducing the expression of GPX4 [40].

Epigenetic changes, including DNA methylation, histone modification, and chromatin remodeling, have been considered to be closely associated with cancer progression and reflect tumor phenotypes [105]. Mao et al. reported that CuB can reduce DNA methyltransferase (DNMT1, DNMT3a, DNMT3b) levels in rectal cancer cells by upregulating BTG3 and inducing promoter demethylation [36]. Shukla et al. also confirmed that CuB-mediated inhibition of DNMTs and HDAC in H1299 cells activates key tumor-associated genes (e.g., CDKN1 and CDKN2A), thereby suppressing cell growth while inducing apoptosis [20].

Drug resistance remains a major hurdle in cancer therapy. Key resistance mechanisms are as follows [106]: overexpression of ATP-binding cassette (ABC) transporters, including P-glycoprotein (P-gp), multidrug resistance-associated protein 1 (ABCC1), and breast cancer resistance protein (ABCG2), promotes drug efflux; increased redox proteins inactivate chemotherapeutic agents; and dysregulation of anti-apoptotic genes (e.g., Bcl-2 and MDM2) enables uncontrolled cancer cell proliferation, enhancement of DNA repair capabilities in cancer cells, and the generation of a tumor microenvironment (TME) that is resistant to the damage inflicted by chemotherapy drugs. CuB can be used simultaneously with a variety of anticancer drugs to exert a synergistic anticancer effect, and it has been demonstrated to promote the apoptosis of drug-resistant cancer cells, such as cisplatin-resistant gastric cancer cells [35], multidrug-resistant human hepatocellular carcinoma cells [107], and paclitaxel-resistant human ovarian cancer cells [108]. The mechanisms, which mainly involve inhibiting the expression of P-gp protein and reversing the increased expression of anti-apoptotic proteins, are related to mitochondrial dysfunction and a reduction in energy supply.

## 6. The Application of Cucurbitacin B

CuB was first used in combination with cucurbitacin E (that is, cucurbitacin tablets) as an adjuvant drug for the treatment of chronic hepatitis and liver cancer [109,110]. However, due to the nonspecific toxicity of cucurbitacin, high concentrations of CuB can inflict damage on normal cells. Moreover, CuB is a hydrophobic component with limited solubility in water, which restricts its bioavailability. To address this issue, researchers have been continuously improving the application potential of CuB through methods such as structural modification, combination therapy, and delivery via nanomaterials.

### 6.1. Individual Treatment

Cucurbitacin tablets were applied in clinical practice as early as the 1970s and 1980s. Cucurbitacin tablets have been in clinical use since the 1970s–1980s. Studies indicate that, for elderly patients with primary liver cancer, the short-term efficacy of oral cucurbitacin tablets reaches 89.4%. When comparing the long-term curative effects, the 2-year survival rate of the observation group (68.4%), the average overall survival time (21.5 ± 2.8 months), and the average progression-free survival time (17.4 ± 1.6 months) were notably higher than those of the control group [111]. However, clinical data indicate that taking cucurbitacin tablets can cause reactions such as discomfort in the upper abdomen, along with symptoms such as dizziness, diarrhea, nausea, and liver pain. As a consequence, a rising number of scholars are concentrating on elucidating the mechanism underlying CuB’s anticancer efficacy.

**Figure 3:**
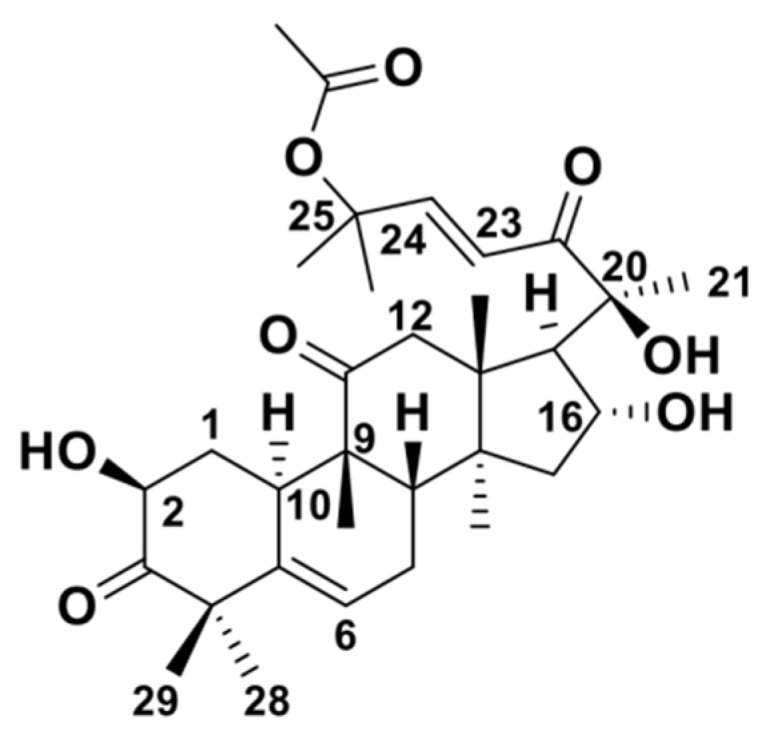
The structure of cucurbitacin B.

The structure of a compound forms the basis of its pharmacological effects. For example, cucurbitacin D demonstrates potent anticancer activity against multiple human cancer cell lines, but its 2-O-glucoside derivative does not [112]. Numerous studies have shown that the CuB derivatives obtained through structural modification are characterized by high efficiency and low toxicity, which significantly broadens their therapeutic window. For example, the introduction of a NO donor at the 16-OH position of CuB (Figure 3) confers heightened cytotoxicity against HepG-2 cells, thereby enhancing the tumor-targeting selectivity of CuB [113]. Suebsakwong et al. modified CuB at the 2-OH position, resulting in a derivative that was less toxic to normal cells while preserving the anti-breast cancer activity of CuB [114].

### 6.2. Combination Therapy

In clinical settings, monotherapy is inadequate for complex diseases. Thus, combination therapies using multiple drugs are commonly used for better efficacy. Thoennissen et al. demonstrated a synergistic antiproliferative effect of CuB–gemcitabine on pancreatic cancer cells [2]. In the in vitro studies by Aribi et al., CuB in combination with gemcitabine inhibited the proliferation of MDA-MB-231 breast cancer cells and significantly reduced the tumor volume compared with monotherapies with the tested chemicals [115]. Additionally, CuB combined with other drugs can increase drug sensitivity in tumor cells, thus serving as an adjuvant to reverse drug resistance. Cisplatin (PtCl_2_(NH_3_)_2_), a cytostatic chemotherapy drug, is used in the treatment of various types of malignant tumors. However, the efficacy of cisplatin treatment was significantly reduced by the drug resistance of malignant tumors. The study by El-Senduny et al. showed that CuB can serve as a chemosensitizer for cisplatin-resistant cell lines, enhancing the growth-inhibitory effects of cisplatin on cancer cells [116]. The same results were also observed in combinations of CuB with paclitaxel and curcumin [107,108].

### 6.3. Nanomaterial Delivery

Besides the above, more researchers are using NPs (e.g., galactosylated solid liposomes [117], exosome-loaded polymeric micelles [118], and cancer cell membrane-cloaked polydopamine NPs [54]) as CuB carriers. Liposomes are common delivery carriers, featuring good biocompatibility, broad-spectrum drug adaptability, high bioavailability, and enhanced drug stability. Exosomes, secreted by diverse cell types (such as cancer cells, immune cells, stem cells, and endothelial cells), play a crucial role in cell–cell communication [119]. Wang et al. developed an exosome-mimicking sequential bioactivatable prodrug nanoplatform (EMPCs), co-encapsulating ROS-responsive paclitaxel linoleate conjugate (PTX-S-LA) and CuB in polymeric micelles and modifying them with exosome membranes (EMs) [118]. This nanomaterial amplifies prodrug bioactivation, prolongs its residence in blood circulation, targets homotypic tumor cells, enhances tumor penetration, and inhibits metastasis by clearing CTCs and regulating the FAK/MMP signaling pathway. Moreover, the discovery and utilization of plant-derived nanovesicles (PDNVs) have further promoted the development of exosomes. PDNVs can be produced on a large scale, have cross-kingdom effects, and possess certain targeting properties. Chen et al. loaded CuB into cucumber-derived outer membrane vesicles that could be taken up by A549 cells to inhibit their proliferation via STAT3 phosphorylation suppression, ROS generation enhancement, cell cycle arrest promotion, and caspase activity increase [120].

## 7. Summary and Outlook

Great progress has been made in researching the anticancer effects of CuB, a tetracyclic triterpenoid from Cucurbitaceae plants. CuB can induce cancer cell apoptosis by inhibiting pathways such as JAK/STAT and PI3K/Akt and the expression of apoptosis-related proteins; arrest the cell cycle at the G2/M or S phase, impeding cancer cell proliferation; and induce autophagy via ROS production and DNA damage and suppression of the AKT/mTOR pathway. In the context of cell migration and invasion, CuB is capable of remodeling the cytoskeleton, suppressing EMT, and affecting multiple signaling pathways. Consequently, it attenuates the migratory and invasive capabilities of cancer cells and reduces the risk of tumor metastasis. Moreover, CuB plays crucial roles in anti-angiogenesis, regulating metabolic reprogramming, enhancing antitumor immune responses, inducing pyroptosis and ferroptosis, and causing epigenetic changes, thereby inhibiting tumor growth and development. Furthermore, as can be seen from Table 2, most of the current research is mainly focused on the cellular level, with relatively few in vivo studies. Moreover, different doses induce different antitumor mechanisms. For example, in the treatment of breast cancer, low doses (18–50 nM) have been mostly verified to activate the HER2-integrin signaling pathway, and are more inclined to target signaling pathways related to cell adhesion and growth factor receptors. In contrast, higher doses can activate ROS to induce DNA damage and autophagy. In addition, some signaling pathways, such as JAK/STAT, are targeted by CuB in various types of cancers, including neuroblastoma, prostate cancer, and lung cancer. This implies that the JAK/STAT pathway is a common and crucial regulator of the growth and survival of cancer cells in different cancer types. Targeting this pathway with CuB may be a broad-spectrum anticancer strategy. In melanoma, the Gα13/RhoA/PKA/VASP pathway is specifically affected. This may be due to the unique biological characteristics. Understanding these unique pathways is helpful for developing more targeted therapeutic methods for specific cancers.

Early cucurbitacin tablets had some efficacy in liver cancer treatment and improved patient survival but caused diverse adverse reactions due to the nonspecific cytotoxicity of CuB; therefore, generating high-efficiency and low-toxicity derivatives through structural modification, achieving synergistic effects and enhancing drug sensitivity via combination therapy (which reduces CuB dosage), and improving its bioavailability and targeting ability through nanomaterial-based delivery system have opened up new avenues for the clinical application of CuB. Nanocarrier-based systems have emerged as a promising solution to address the use issues of CuB. However, different types of nanomaterials each have their own advantages and disadvantages. Liposomes exhibit good biocompatibility, and there are FDA-approved precedents, but their application is limited by low drug-loading capacity and poor stability [121]. Inorganic nanoparticles or organic nanoparticles (e.g., mesoporous silica, metal-organic frameworks) offer the advantage of high drug-loading capacity, but they also have the problem that heavy metals or non-degradable materials can accumulate continuously in the body, resulting in clinical translation [122,123]. Existing studies have shown that surface modification with pH-sensitive polymers or targeting peptides can enhance the accumulation of nanoparticles at tumor sites [124,125], and replacing traditional PLGA or inorganic carriers with natural polymers (such as hyaluronic acid, sodium alginate) or degradable synthetic materials (such as polycaprolactone) can reduce long-term toxicity [126]. Evidently, the development of intelligent carriers is increasingly emerging as a future trend in the clinical translation research of CuB.

Despite remarkable progress in the research of antitumor CuB, there is still a substantial scope for further investigation. For instance, the antitumor mechanism and targets of CuB involve multiple pathways, yet the interactions among them have not been fully elucidated. In addition, there are a few studies on the interaction between CuB and the immune system, including the immune-cell activation pathways and the immune-microenvironment regulation mechanisms. It is worth noting that cucurbitacin B (CuB) affects glucose, lipid, and amino acid metabolism and can inhibit tumor progression by altering metabolic reprogramming. The literature has shown that CuB can inhibit glycolysis in HuCCT1 cells by regulating the Akt/mTOR pathway, thereby reducing lactate secretion. Lactate can suppress the function and survival of T cells and NK cells, facilitating tumor immune evasion [127,128]. Whether the immunomodulatory effects of CuB interact with its metabolic regulatory effects merits further investigation. However, complex physiological processes—such as in vivo drug ADME (absorption, distribution, metabolism, excretion), dynamic crosstalk between immune and stromal cells in the tumor microenvironment, and the impact of systemic circulation on drug stability—complicate translation [129]. For example, the efficient tumor targeting of CuB via nanocarriers observed in vitro may be severely compromised in vivo due to reticuloendothelial system clearance or abnormal tumor vasculature [130]. Thus, in vivo validation of mechanisms and delivery strategies is critical to bridging preclinical and clinical research.

Advancements in biotechnology, such as CRISPR technology and single-cell RNA sequencing, are poised to offer novel perspectives and methodologies for CuB research. By leveraging these tools, we can delve deeper into antitumor mechanisms, identify potential biomarkers, and uncover novel insights into CuB.

## 8. Conclusions

CuB has broad-spectrum antitumor effects on many types of cancer and is involved in multiple mechanisms. Because of the relatively low toxicity of the cucurbitacin family, it has broad application prospects in tumor treatment. However, its non-selective toxicity, poor water solubility, and low bioavailability limit its clinical application. Therefore, researchers often combine CuB with other drugs, modify its structure, or use nanomaterial delivery or other methods to reduce its toxicity, improve its bioavailability, and thus enhance its antitumor effect. Nevertheless, further research is needed to explain the mechanisms underlying the different sensitivities of various cells to cucurbitacin B. In particular, CuB also has an impact on the tumor immune microenvironment, but the specific mechanisms of its interaction with the immune system need to be further explored.

## Figures and Tables

**Table 1:** Physical and chemical properties of CuB.

Name	CuB
Molecular Formula	C_32_H_46_O_8_
Molecular Weight	558.7029
Form	Powder
Colour	White
Density	1.0953
Melting Point	180–183 °C
Boiling Point	699.3 ± 55.0 °C (760 mmHg)
Refractive Index	1.4900
Solubility	DMSO:10 mg/ml
Flash Point	218.7 °C
Vapor Pressure	1.3 × 10^−22^ mmHg (25 °C)
Polar Surface Area	138.20000
LogP	3.49930
pKa	12.60 ± 0.29
Maximum Wavelength	290 (in ethanol solution)
Optical Rotation	D25 + 88° (c = 1.55, in ethanol solution)
Storage Conditions	Powder: −20 °C (3 years), 4 °C (2 years); Solution: −80 °C (6 months), −20 °C (1 month)

**Table 2:** The main pharmacological effects of CuB.

Cancer	Subjects	In Vitro Dosage	In Vivo Dosage	Mechanisms	Ref.
Breast cancer	Hela cell	3 μM		Inhibit the mTOR/p70S6k/4EBP1 and MEK/ERK signaling pathways by suppressing HIF-1 activation.	[7]
	BALB/c nude		1 and 5 mg/kg; oral		
	mice				
	MDA, MB-231, SKBR3, MCF-7 and 4T-1 cell	18–50 nM		Inhibit the expression of ITGA6B4 and induce the expression of ITGB1 and ITGB3, promoting the apoptosis of breast cancer cells by inhibiting the HER2-integrin signaling pathway.	[8]
	MCF-7 cell	0–200 nM		Induce DNA damage and autophagy by increasing the level of ROS.	[9]
	MDA-MB-231 and 4T1 cell	12.01 μM, 80 nM		Downregulate the VEGF/FAK/MMP signaling pathway to inhibit metastasis and angiogenesis.	[10]
	Balb/c mice		0.1 and 0.25 mg/kg; i.p.		
	MDA-MB-231 and SKBR-3 cell	0–125 μM		Mediate the biomechanical properties of breast cancer through the RAC1/CDC42/RhoA signaling pathway, thereby inhibiting cell migration and invasion.	[11]
	Balb/c nude mice		0.5 mg/kg; i.p.		
Breast cancer and osteosarcoma	Hela, MCF-7 and U2OS cell	12.2, 22.93, 17.07 nM		Interact with the cytoskeleton by affecting actin filaments through depolymerization and aggregation, and induce cell cycle arrest.	[12]
Osteosarcoma	HOS, and 143B	0–140 nM		Inhibit M2 macrophage differentiation by inhibiting the PI3K/Akt pathway.	[13]
	Balb/c nude mice		1 mg/kg; i.p.		
Melanoma	A375 and B16F10 cell	0–1 μM		Induce the aggregation of actin and the formation of filamin-actin rods via the Gα13/RhoA/PKA/VASP pathway.	[14]
	CRMM2, CM-AS16, CRMM1 and CM2005.1 cell	0.15, 0.08, 0.24 and 0.38 μM		Induce cell cycle arrest by inhibiting the GRP78/eFOXM1/eKIF20A pathway.	[15]
	NCG mice		1 mg/kg; oral		
Neuroblastoma	SH-SY5Y cell	0–128 μM		Inhibit the growth and proliferation of SHSY5Y human neuroblastoma cells by inhibiting the JAK2/STAT3 pathway and activating the MAPK pathway.	[16]
Prostate cancer	LNCaP and PC-3 cells	0–0.3 μM		Induce apoptosis of prostate cancer cells through the ROS-dependent ACLY signaling pathway.	[17]
	nude mice		0.1 μmol; oral		
	BPH-1 cell	0–200 nM		Inhibit prostate cell proliferation by activating p53/MDM2 signaling cascade and downregulating COX-2 expression.	[18]
Lung cancer	A549	0–200 nM		Induce cell DNA damage by increasing the formation of ROS, and then induce cell-cycle arrest.	[19]
	A549, H1299 and H1650 cell	0–860 nM		Cause the upregulation of TSGs and the downregulation of TPG through changes in histone modification and promoter methylation, thereby inhibiting the growth and inducing apoptosis of NSCLC cells.	[20]
	A549, H1299 and H23 cell	0–200 nM		Inhibit the metastatic ability of NSCLC by suppressing the Wnt/β-catenin signaling axis.	[21]
	PC9 cell	0–50 μM		Improve the resistance of NSCLC to gefitinib by regulating the miR175p/STAT3 axis to reduce the protein level and phosphorylation of STAT3, inhibit proliferation, and promote cell apoptosis	[22]
	PC3 cell	0–25 µM		Induce apoptosis and cell cycle arrest in PC3 cells by downregulating of JAK/STAT signaling cascade.	[23]
	A549 cell	0–20 nM		Inhibit TGF-β1-induced EMT in A549 cells and gefitinib-resistant A549 cells, and inhibit cell migration and invasion by reducing the production of ROS and the PI3K/Akt/mTOR signaling pathway.	[24]
	C57BL/6J mice		0.25 and 0.5 mg/kg		
	A549 cell	0–0.9 μM		Suppress the proliferation and induce the apoptosis of lung cancer cells by inhibiting the IL-6/STAT3 pathway through the lncRNA XIST/miR-let-7c axis.	[25]
	A549, H1299, H1975, H820 and 16-HBE cell	0–100 nM		Suppress the growth and invasion of GR NSCLC cells by inducing the lysosomal degradation of EGFR and downregulating the CIP2A/PP2A/Akt signaling axis.	[26]
	Balb/c nude mice		0.5 mg/kg		
	A549 cell	0–1000 nM		Induce pyroptosis by binding to TLR4, thereby inhibiting tumor growth.	[27]
	C57BL/6 mice		0.25, 0.5, and 0.75 mg/kg; i.p.		
	H358, A549, H23, H1650 and PC9 cell	0–100 μM		Induce ferroptosis and the proliferation of non-small cell lung cancer by inhibiting the activation of STAT3.	[28]
Liver Cancer	BEL-7402 cell	0–100 nM		Induce DNA damage mediated by ROS, and then activate PTEN to promote protective autophagy.	[29]
	HepG2 cell	0–500 nM		Inhibit the growth and proliferation of CD133+ HepG2 cells by inhibiting the JAK2/signal transducer and activator of transcription-3 signaling pathway to induce cell cycle arrest.	[30]
	Balb/c nude mice		0.75 mg/kg; oral		
	Huh7 cell	0–100 μM		Block the m^6^A mRNA connection of IGF2BP1, thereby activating tumor immune microenvironment (TIME).	[31]
	Balb/c mice		1 and 5 mg/kg; i.p.		
	HepG2 cell and HUVEC cell	0–100 nM		Induce the mitochondrial apoptosis pathway to trigger apoptosis in HUVEC cells and inhibit the activity of VEGFR2, thereby suppressing angiogenesis.	[32]
	FVB/N mice		2 mg/kg; oral	Affect lipid metabolism, amino acid metabolism, and glucose metabolism by changing the Akt/mTORC1 signaling pathway, thereby reducing tumor progression.	[33]
	Huh7, Hep3B and Hepa1/6	0–30 μM		Activate the ATM-dependent p53-p21-CDK1/CHK1/CDC25C signaling pathway, induce DNA damage, and consequently lead to cell cycle arrest.	[34]
	Balb/c nude mice		0.5 and 1 mg/kg; i.p.		
Gastric cancer	SGC7901 cell	0–600 nM		Induce autophagy and apoptosis in human cisplatin-resistant gastric cancer cells by inhibiting the CIP2A/PP2A/mTORC1 signaling axis.	[35]
Colorectal cancer	SW480 and Caco-2 cell	0–50 μM		Inhibit the proliferation of colorectal cancer cells and induce apoptosis by regulating the demethylation of the BTG3 promoter.	[36]
	CT-26 and HCT116 cell	0–2400 nM		Regulate TAMs through JAK2/STAT3 signaling pathway to inhibit the growth and metastasis of colon cancer cells.	[37]
	C57BL/6 and Balb/c mice		0.5 and 1 mg/kg; i.p.		
Cholangiocarcinoma	KKU-100 cell	0.1–40 μM		Induce the intrinsic mitochondrial apoptosis pathway in CCA cells by inhibiting Fak-mediated PI3K/Akt.	[38]
Lymphoma	BCBL-1, BC-1, GTO and TY-1 cell	0–50 nM		Induce apoptosis of BCBL-1 cells by activating caspases, and cause cell cycle arrest of BCBL-1 cells by inducing actin aggregation and inhibiting the level of p-filamin.	[39]
	Balb/c mice		0.5 mg/kg; i.p.		
Nasopharyngeal carcinoma	CNE1 cell	16 nM		Induce ferroptosis to cause cell death by increasing lipid peroxidation and decreasing the expression of GPX4.	[40]
	Balb/c mice		0.5 and 1 mg/kg; i.p.		
Ovarian cancer	A2780, OV2008, C13, and A2780-DDP cell	0–1 μM		Inhibit PI3K/Akt/mTOR signaling pathway, thereby inhibiting the proliferation of cisplatin-resistant ovarian cancer cells, inducing DNA damage, activating cGASA, and activating immune regulation.	[41]
	Balb/c nude mice		0.25, 0.5 and 1 mg/kg; i.p.		
Oral cancer	HOK and DOK cell	0–120 nM		By activating the SLC7A11/mitochondrial oxidative stress pathway to induce ferroptosis to prevent the progression of malignant tumors.	[42]
	C57BL/6 mice		0.5 and 1 mg/kg; i.p.		
Esophageal cancer	Het1A, TE-1, KYSE410, ECA109 and KYSE150 cell	0–0.4 μM		Weakening the JAK/STAT3 pathway by inhibiting KIF20A expression to inhibit the progression of ESCA.	[43]
